# A review of the pharmacogenomics of buprenorphine for the treatment of opioid use disorder

**DOI:** 10.20517/jtgg.2020.35

**Published:** 2020-07-30

**Authors:** Hemanuel Arroyo Seguí, Kyle Melin, Darlene Santiago Quiñones, Jorge Duconge

**Affiliations:** 1 School of Pharmacy, University of Maryland, Baltimore, MD 21201, USA.; 2 Department of Pharmacy Practice, School of Pharmacy, University of Puerto Rico Medical Sciences Campus, San Juan, PR 00936, USA.; 3 Department of Pharmaceutical Sciences, School of Pharmacy, University of Puerto Rico Medical Sciences Campus, San Juan, PR 00936, USA.

**Keywords:** Pharmacogenomics, pharmacogenetics, buprenorphine, opioid use disorder, opioids, personalized medicine, medication-assisted treatment, buprenorphine/naloxone

## Abstract

As the opioid epidemic continues to grow across the United States, the number of patients requiring treatment for opioid use disorder continues to climb. Although medication-assisted treatment presents a highly effective tool that can help address this epidemic, its use has been limited. Nonetheless, with easier dosing protocols (compared to the more complex dosing required of methadone due to its long and variable half-life) and fewer prescribing limitations (may be prescribed outside the setting of federally approved clinics), the increase in buprenorphine use in the United States has been dramatic in recent years. Despite buprenorphine’s demonstrated efficacy, patient-specific factors can alter the response to the medications, which may lead to treatment failure in some patients. Clinical characteristics (sex, concurrent medications, and mental health comorbidities) as well as social determinants of health (housing status, involvement with the criminal justice system, and socioeconomic status) may impact treatment outcomes. Furthermore, a growing body of data suggests that genetic variations can alter pharmacological effects and influence therapeutic response. This review will cover the available pharmacogenomic data for the use of buprenorphine in the management of opioid use disorders. Pharmacogenomic determinants that affect opioid receptors, the dopaminergic system, metabolism of buprenorphine, and adverse events are discussed. Although much of the existing data comes from observational studies, clinical research is ongoing. Nevertheless, the development of pharmacogenomic-guided strategies has the potential to reduce opioid misuse, improve clinical outcomes, and save healthcare resources.

## INTRODUCTION

The United States (US) has seen a surge in prescription and illicit use of opioids in the last twenty years, creating an unprecedented healthcare and socioeconomic crisis. Approximately 2 million people in the US have opioid use disorders (OUD) resulting from prescription usage alone^[1]^. This life-threatening chronic brain disease is associated with a significant increase in early death, resulting from overdose, trauma, suicide or infectious diseases (e.g., Hepatitis C, HIV/AIDS)^[2]^. From 1999 to 2018, approximately 450,000 people died in the US due to opioid overdoses^[3]^. Healthcare costs, loss of productivity, and criminal involvement creates substantial socioeconomic burden^[4]^. The Centers for Disease Control and Prevention estimate costs of more than $78 billion per year in the US from prescription opioid misuse, and others have estimated an additional loss of $50 billion from heroin use^[5,6]^.

The prolonged and repeated administration of opioids over time causes lasting effects on neuronal structure and function^[4]^. The 5th edition of the Diagnostic and Statistical Manual of Mental Disorders defines OUD as a problematic pattern of misuse resulting in clinically significant impairment or distress^[7]^. OUD can be effectively managed using medication-assisted treatment (MAT), a combination of pharmacological and behavior-based interventions such as, cognitive-behavioral therapy, contingency plans, 12-step programs and support groups^[2]^. Evidence strongly recommends the use of medications in all patient groups, including adolescents, pregnant women and older adults^[4,8–14]^. Pharmacotherapy can reduce morbidity and mortality by restoring brain function, supporting opioid abstinence and reducing risk for overdose death^[4,15,16]^. OUD treatment can also lower the risk of blood-borne infections and help patients recover social functionality, easing reintegration into their communities^[17–19]^.

After initial detoxification (i.e., opioid withdrawal management), patients should be introduced to maintenance therapy with MAT. FDA-approved medications for maintenance therapy include methadone, buprenorphine and extended-release naltrexone^[2,4]^. All three medications target receptors within the opioid system and work by reducing opioid cravings and/or decreasing the response to future drug use^[4]^. Due to their direct agonistic effect on opioid μ-receptors, methadone and buprenorphine are also indicated for symptomatic relief of acute opioid withdrawal^[2]^. Despite the availability of these life-saving medications, most patients do not receive adequate treatment^[4]^. Social barriers and policy regulations limit the number of patients who receive pharmacotherapy, especially in underserved communities (e.g., African Americans, Hispanics). Methadone can only be dispensed in federally approved clinics [i.e., opioid treatment programs (OTPs)], restricting access for many patients^[2]^. Buprenorphine can be prescribed in office-based settings, but providers require completion of an 8-hour training course, and obtain a waiver from the Drug Enforcement Administration^[20]^. About 2%−3% of providers in the US have such waivers^[4,21]^.

Furthermore, patient-specific factors can alter the response to these medications, which may lead to treatment failure in some patients^[4,22,23]^. Clinical characteristics such as sex, concurrent medications, and mental health comorbidities have all been shown to affect patient variability in response to pharmacotherapy^[24–26]^. Social determinants of health such as housing status, involvement with the criminal justice system, and socioeconomic status may also impact treatment outcomes^[4,27,28]^. Lastly, genetic variations can alter pharmacological effects and influence therapeutic response^[29–31]^. Pharmacogenomic studies evaluate genotypic-phenotypic associations and their potential impact on pharmacokinetic (e.g., metabolic function, dosing requirements) and pharmacodynamic (e.g., receptor activity, treatment efficacy) parameters. In recent years, pharmacogenomic research for OUD has accelerated.

To date, the majority of pharmacogenomic studies for OUD treatment have focused on methadone use, where a number of relevant pharmacogenes have been identified. Methadone is a full agonist at the μ-opioid receptor, with antagonistic activity at the N-methyl-D-aspartate (NMDA) receptor^[20]^. This NMDA antagonism can potentially cause cardiac toxicity, especially in patients with an elevated QTc interval. Regarding pharmacogenes, *CYP2B6* variants (e.g., *4, *6, *9 alleles) have been associated with alterations in metabolic rate and plasma methadone concentrations, which could determine dosing requirements^[32–34]^. *OPRD1* variants (e.g., rs678849) have been shown to impact treatment outcomes in African American patients, while *OPRM1* variants (e.g., rs10485058, rs3192723) may impact efficacy in European and Asian patients^[31,35]^. *UGT2B7* variants (e.g., rs6600879, rs4554144) may also determine severity of opioid withdrawal in some populations^[36]^. The evidence related to the pharmacogenomics of methadone has recently been reviewed elsewhere and, therefore, will not be further discussed here^[37,38]^.

Buprenorphine is a high-affinity, μ-receptor partial agonist^[20]^. It also possesses antagonistic activity on κ-receptors, and weak agonist activity on δ1-receptors. Partial agonism leads to effective analgesic effects and abatement of withdrawal symptoms while reducing the risk of respiratory depression observed with methadone and other opioids^[20]^. Additionally, due to its effects on κ-receptors, clinical data have suggested that buprenorphine may possess antidepressant benefits, potentially representing a better option for patients with comorbid depression^[39,40]^. Buprenorphine has also been co-formulated with naloxone as a sublingual film or tablet to discourage intravenous administration, which results in the immediate onset of euphoric effects^[20]^. Naloxone is a high-affinity μ-receptor antagonist that displaces other opioid ligands, essentially blocking and reducing their pharmacological effects (e.g., euphoria, respiratory depression). With easier dosing protocols (compared to the more complex dosing required of methadone due to its long and variable half-life) and fewer prescribing limitations (may be prescribed outside the setting of federally approved clinics), the increase in buprenorphine use has been dramatic in recent years^[21]^. The number of patients receiving therapy from non-OTP facilities increased from 1,670 in 2004 to 54,488 in 2015, an increase of three thousand percent. However, pharmacogenomic studies including buprenorphine have lagged behind those of methadone. Understanding how to tailor drug treatment in OUD is essential to reducing opioid misuse, improving treatment adherence, and saving healthcare resources. This review will cover the available pharmacogenomic data for the use of buprenorphine in the management of OUDs.

## PHARMACOGENES: OPIOID RECEPTORS

Activity on all three G-protein coupled receptors of the opioid receptor family (μ, δ and κ), differentiates buprenorphine from other opioid-based medications^[41]^. These receptors are structurally and functionally related, but exhibit variations in ligand affinity and cellular distribution^[42]^. The main effects of opioids (i.e., analgesia, reward, adverse effects) are mediated through μ-receptor binding, while δ- and κ-receptors help modulate these effects via different biological mechanisms. Alterations in opioid receptor expression and function (i.e., genetics), may impact substance dependence risk and influence treatment response^[31,42]^.

### μ-Opioid receptor gene (*OPRM1*)

As the primary target in opioid therapeutics, it is reasonable to assume that activity on μ-receptors plays a crucial role in opioid dependence, and that variations in the genes encoding these receptors may impact clinical outcomes. Although *OPRM1* variants (e.g., rs9479757G>A) have been associated with heroin dependence risk, pharmacogenomic analyses of *OPRM1* variants have demonstrated conflicting results in OUD treatment response^[43]^.

Data from the randomized clinical trial Starting Treatment With Agonist Replacement Therapies (START), has been used to perform several pharmacogenomic analyses of OUD treatment outcomes^[44]^. Genotypic information was available for 60% (*n* = 764/1,267) of the patient population, which was primarily composed of European Americans (*n* = 599), with few African Americans (*n* = 79) and other ethnicities (*n* = 96)^[29]^. Over a 24-week period, participants treated with methadone (*n* = 364, 66% male) or buprenorphine/naloxone (*n* = 410, 71% males) were submitted to weekly urine drug screens. From the several pharmacogenomic studies conducted from this trial, only two evaluated *OPRM1* variants^[29,30]^. No significant results were observed between variants and buprenorphine response in either study. One study compared the genotypes of all participants at the *OPRM1* variant rs1799971, with dropout rate and dosing requirements^[29]^. The other used a sample of European Americans (*n* = 582) and analyzed the effects of single-nucleotide polymorphisms (SNPs) in an untranslated region (3’) of *OPRM1,* on the number of opioid-positive urine tests^[30]^. Although no differences were found with buprenorphine, this second study did show an association between the *OPRM1* variant rs10485058 and methadone-treated patients. AA genotypes at this locus were less likely to have opioid-positive urine tests than carriers of the G-allele (RR = 0.76, *P* = 0.0064).

In addition to OUD trials, one study evaluated the effect of the *OPRM1* 118A>G (rs1799971) polymorphism on the analgesic effect of buprenorphine^[45]^. The study was conducted in a Spanish population (*n* = 93, 77% male) with critical limb ischemia who were hospitalized for revascularization and treated with transdermal buprenorphine for pain. No significant association was found between rs1799971 and the analgesic effect of buprenorphine, although significant findings were observed with other pharmacogenomic variables, which will be discussed later in this review. On the other hand, a study evaluating an individualized opioid deprescription program with buprenorphine, did show a significant association between the same *OPRM1* variant rs1799971 and morphine equivalent daily dose (MEDD) requirements^[46]^. Participants included patients of European ancestry (*n* = 88, 64% female) with chronic non-cancer pain, who were using opioids long-term (> 6 months) and were diagnosed with prescription opioid dependence. Interventions involved a slow-tapering process, with a 30% reduction in opioid dose and rotation to buprenorphine and/or tramadol. Carriers of the rs1799971 variant required significantly higher MEDD (*P* < 0.05) for analgesic control and prevention of withdrawal symptoms. No associations were found between *OPRM1* and program response.

### δ-Opioid receptor gene (*OPRD1*)

Multiple studies have demonstrated the impact of δ-Opioid receptor gene (*OPRD1)* genetic variants on substance dependence risk^[42,47]^. As for OUD treatment, a series of SNPs located on *intron 1*, may be predictive of outcomes in some patient populations receiving buprenorphine. A patient cohort from the START trial was used to evaluate the effects of *OPRD1* variants on opioid abstinence, which was measured by urine drug screens^[31]^. This sample was primarily composed of European Americans (*n* = 566, 68% male) and some African Americans (*n* = 77, 69% male). Although no significant differences were observed in the amount of opioid-positive tests when comparing methadone and buprenorphine, the rs678849 variant of *OPRD1* was significantly associated with treatment outcomes in African Americans for both medications. In the buprenorphine group, African Americans with the CC genotype (*n* = 24) were more likely to have opioid-positive drug screens than CT/TT genotypes (*n* = 17, RR = 2.17, *P* = 0.008). The opposite was observed for the methadone group, as African Americans with the CC genotype (*n* = 21) were less likely to have opioid-positive drug screens than carriers of the T-allele (*n* = 15) (*R* = 0.52, *P* = 0.001). No significant results were shown in European Americans for any *OPRD1* genetic variant. The researchers concluded that for African Americans, matching rs678849-related genotypes to pharmacotherapy could improve treatment efficacy overall^[31]^. Estimates have indicated that half of all African Americans may possess the CC genotype^[37]^. A follow-up analysis was performed to replicate the results of this study using an independent cohort of African Americans^[48]^. This replication study confirmed that the rs678849 genotypes were related to buprenorphine outcomes: CC genotype was more likely to have opioid-positive tests than CT/TT genotypes (RR = 1.69, *P* = 0.021). This analysis was unable to confirm the previously identified association between the C-allele and treatment success in the methadone group. Of note, the methadone group in this cohort was relatively small with only 22 participants.

In addition to the *OPRD1* variant rs678849, two other intronic SNPs have been identified as clinically relevant in buprenorphine therapeutics. Using the European American sample from the START trial, a pharmacogenomic analysis was conducted to evaluate sex-specific differences on clinical outcomes^[49]^. *OPRD1* variants were compared between males and females undergoing methadone or buprenorphine/ naloxone treatment. No significant interactions were observed for males on either treatment, nor females on methadone. However, in females treated with buprenorphine (*n* = 81), genotypes at rs581111 and rs529520 predicted therapeutic response. During the 24-week treatment period, females with a GG genotype at rs581111 were more likely to have opioid-positive tests than AA/AG genotypes combined (RR = 1.72, *P* = 0.031). At rs529520, females with the AA genotype were more likely to have opioid-positive tests than females with the CC genotype (RR = 1.65, *P* = 0.025). Outcomes in carriers of the AC genotype were not significantly different from those with the AA genotype. The researchers concluded that genotypes at these two *OPRD1* variants may be useful when considering OUD pharmacotherapy for females, but further validation is warranted. Interestingly, sex and gender differences have previously been shown to impact OUD outcomes, as well as buprenorphine pharmacokinetic and pharmacodynamic parameters^[4,24,50–52]^.

### κ-Opioid receptor gene (*OPRK1*)

κ-Opioid receptors may play an important role in buprenorphine therapeutics, especially for patients with comorbid depression. The antidepressant effects displayed by buprenorphine are related to its antagonistic activity at these receptors^[39,40]^. Uncontrolled psychiatric illnesses can negatively impact OUD treatment outcomes, and thus, a medication that could potentially provide benefits for both disorders would be clinically advantageous^[53,54]^. To date, one study has evaluated *OPRK1* genetic variants on buprenorphine response^[55]^. The patient population was composed of Western Europeans (*n* = 107, 81% male) who had a history of heroin-dependence for 4–7 years. Buprenorphine was administered for a 6-month period, and participants were classified as responders or non-responders on the basis of their clinical outcomes (e.g., relapse, treatment completion). Genotypes at the *OPRK1* 36G>T SNP (rs1051660) were compared between response groups, but no significant differences were observed. Of note, due to the low numbers of TT and GT genotypes observed in the study population, the two genotypes were collapsed, and the comparison was made between GG versus GT/TT (i.e., dominant genetic model). In addition, the small number of patients affected by mental health comorbidities precluded an analysis of the association between gene variants and psychiatric illnesses. Despite the negative finding of this small study, further investigations of this variant in other populations and of other genetic variants influencing the function of the κ-opioid system, are needed to assess the effects of *OPRK1* genetic variants on OUD treatment outcomes. Of note, this study by Gerra *et al*.^[55]^ did find a significant association with a dopamine-related pharmacogene, which will be discussed later.

### Discussion

Preliminary evidence suggests that some opioid receptor genetic variants may be associated with buprenorphine treatment outcomes in OUD. The strongest evidence to date shows an association with variants in intronic *OPRD1* SNPs and treatment efficacy, but race and sex may modulate these effects. The rs678849 variant in particular shows promise as a potential clinical variable that could be useful to direct individuals toward buprenorphine (carriers of CT/TT genotypes) or methadone (carriers of CC genotype) in African Americans. Additionally, the rs581111 and rs529520 variants have the potential for utility among females of European ancestry, especially given the differences observed in the opioid system between sexes. Nonetheless, further research is needed with larger and more diverse populations to better understand the role of these variants and other opioid receptor variants in pharmacogenomics-guided treatments.

## PHARMACOGENES: DOPAMINERGIC SYSTEM

As discussed, buprenorphine acts on κ-receptors via antagonism. These receptors are expressed in dopaminergic neurons, where they modulate the release of dopamine^[40]^. The dopaminergic system has been tied to signaling pathways related to reward, mood, and behavior^[56]^. κ-Receptor agonists inhibit the release of dopamine, which can induce stress and dysphoria^[57]^. Furthermore, substance-dependent states are associated with κ-opioid system overdrive, which may reinforce drug-seeking behaviors due to the high levels of stress. Buprenorphine administration may antagonize these receptors, helping to normalize levels of dopamine in the nucleus accumbens, and potentially improving mood and impulsive behavior tendencies. Other monoamines (e.g., serotonin) may also be involved in these antidepressant effects. To date, several studies have evaluated the relationship between dopaminergic pharmacogenes and the clinical outcomes of buprenorphine therapy.

D2-receptor gene (*DRD2*) dysfunction has been related to drug-seeking behavior, and therefore, genetic variants may influence OUD treatment response^[58]^. Although the *DRD2 TaqI* A1 polymorphism has been associated with poor outcomes among methadone patients, no such association has yet been observed with buprenorphine^[59]^. A retrospective study evaluated the presence of the *DRD2 TaqI* A1 allele in treatment response with methadone (*n* = 46, 57% males) and buprenorphine (*n* = 25, 68% females)^[60]^. The patient population was mostly composed of Australians with European ancestry (88%). No significant associations were found related to buprenorphine dose or response, although methadone patients who were *DRD2 TaqI* A1 carriers experienced less withdrawal symptoms than non-carriers (*P* = 0.04).

The previously mentioned study by Gerra *et al*.^[55]^ also evaluated the presence of different alleles at the dopamine transporter gene (*SLC6A3/DAT1*) on buprenorphine response. As mentioned, participants (*n* = 107, 81% males) were classified as responders or non-responders. Non-responders showed continuous use of heroin, severe psychiatric distress, medication diversion and/or dropout of treatment. A significant difference was observed between groups, as all carriers of the 11-repeat allele were able to complete treatment for the duration of the study, without significant disturbances (*P* = 0.001). In addition, the frequency of the 10-repeat allele was higher in non-responders (65% *vs*. 56%), but statistical significance was not established.

### Discussion

The research that exists to date, regarding the role of dopaminergic pharmacogenes on buprenorphine therapeutics is still limited. The study of the *DRD2 TaqI* A1 allele by Barratt *et al*.^[60]^ was likely underpowered to identify meaningful associations, particularly in the buprenorphine arm. However, their preliminary findings in the methadone group suggest further research is justified. The results from Gerra *et al.*^[55]^ suggest a potential clinical utility in *SLC6A3/DAT1* variants that also warrants further study.

## PHARMACOGENES: METABOLISM

**Figure 1.**
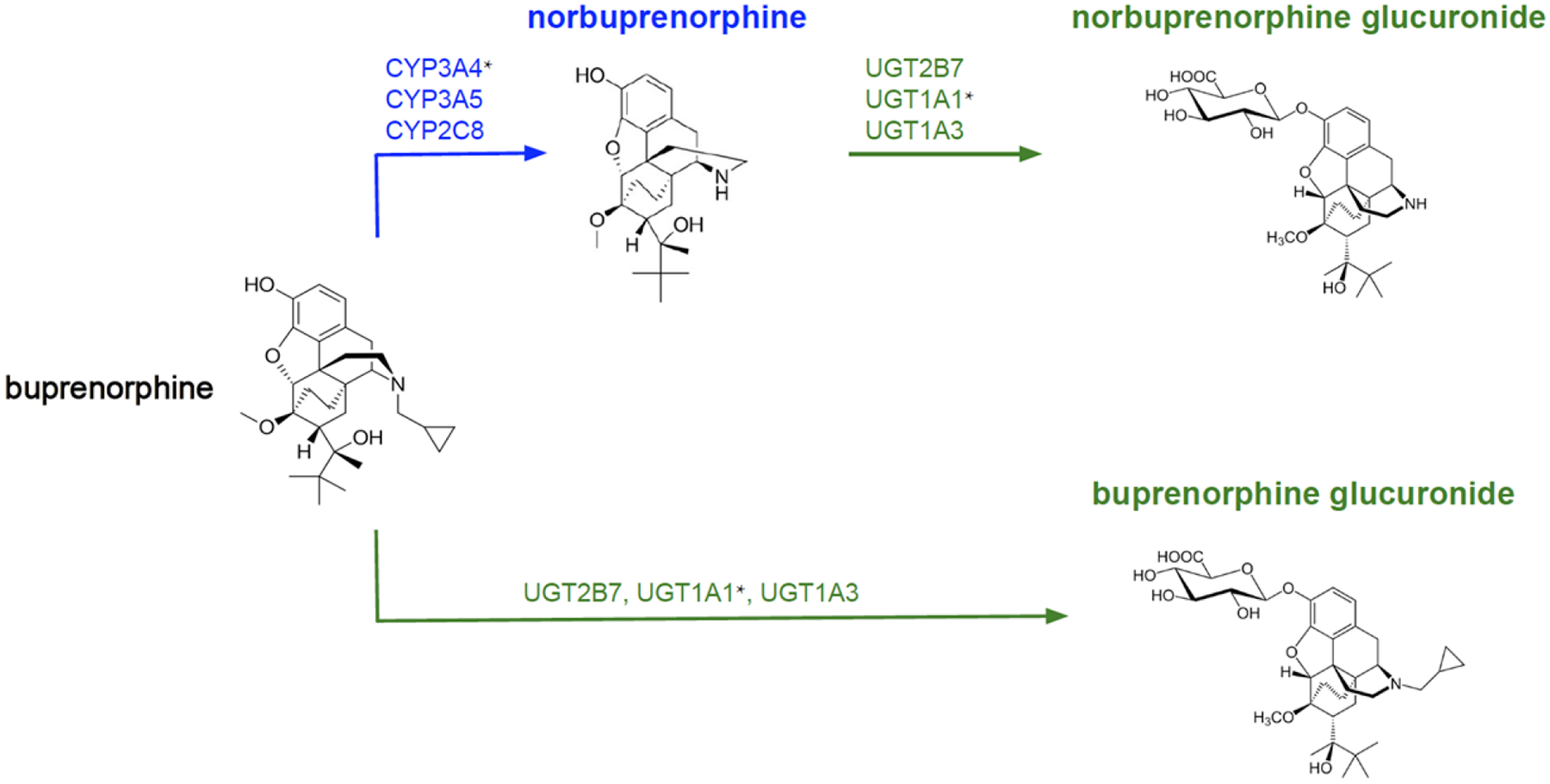
The metabolic pathways of buprenorphine. *Variants of this gene have been found to be clinically relevant

The metabolic rate of drugs can vary between individuals, making dosing adjustments necessary to achieve the desired effects. Buprenorphine is extensively metabolized in the liver via N-dealkylation by cytochrome P450 (CYP450) enzymes, producing nor-buprenorphine, the major active metabolite^[61,62]^. To a lesser extent, both buprenorphine and nor-buprenorphine undergo glucuronidation by UGT [Figure 1]. *CYP3A4* may be considered the primary CYP450 enzyme in the metabolism of buprenorphine, but other enzymes involved have been identified. Using human liver microsomes, a study found that CYP3A4 and CYP2C8 were able to produce the active metabolite in considerable concentrations^[61]^. CYP3A5 and CYP3A7 were also identified as contributors in the metabolic pathway. Approximately 90% of buprenorphine was shown to be metabolized by CYP3A enzymes, and thus, genetic variations due to ancestry and/or sex differences in these enzymes may impact buprenorphine exposure^[63]^. In addition to the CYP450 system, the *UGT2B7* gene has been identified as playing a minor role in buprenorphine metabolism^[64]^. In a study with human liver microsomes, the presence of the *UGT2B7* promoter (G-842A) mutation resulted in higher buprenorphine glucuronidation V_max_ (80% on average) and a higher glucuronidation rate in non-carriers (but not in carriers) of the UGT1A1*28 allele (*P* = 0.0352).

Phenotypic classifications have been developed to categorize functional variants of *CYP3A4*, as poor (PM), intermediate (IM), extensive (EM), or ultrarapid (UM) metabolizers^[65]^. Without dose adjustments, poor metabolizers may have higher than normal plasma levels of buprenorphine, potentially increasing the risk for adverse events. Similarly, ultrarapid metabolizers may have lower plasma levels, which may induce opioid cravings and/or withdrawal symptoms. This particular scenario was observed in a case report involving an African American male undergoing OUD management with buprenorphine/naloxone^[65]^. During every medical appointment, a urine screening was performed to assess adherence and detect the use of unauthorized or illicit substances. This patient had no significant socioeconomic barriers (i.e., married, stable home and employed) and was being treated with a buprenorphine daily dose of 28 mg. For a few years the patient was adherent with no use of other opioids, although occasionally used synthetic cannabinoids. However, following a dosage decrease to 24 mg due to a change in insurance coverage, the patient reported withdrawal symptoms and had multiple recurrences with morphine, methadone, benzodiazepines and synthetic cannabinoids. Pharmacogenomic testing found the patient to have a CYP3A4*1/*1B diplotype, which has been associated with an increased metabolic rate^[66,67]^. Less than a year later, the third-party payer dose-limiting policy was cancelled and the patient went back to 28 mg for one month, and then increased to 32 mg. During the next 4 months with 32 mg, the patient did not use any unauthorized substances. As a CYP3A4*1B carrier, the patient was classified as an ultrarapid metabolizer, consistent with a higher dose requirement.

The same research group then conducted a retrospective cohort study to evaluate *CYP3A4* and *CYP3A5* polymorphisms on buprenorphine response (i.e., dosing, withdrawal and relapse)^[67]^. The patient population was mostly composed of African Americans (*n* = 111/113, 76% males). Participants were classified by their *CYP3A4* phenotype, and the majority of the patient population were ultrarapid metabolizers (82%), carrying at least one *1B allele. Clinical outcomes assessed included withdrawal instances, use of unauthorized substances (i.e., urine drug screens), and dosing comparisons between standard-of-care (SOC) and PGx guided treatment. With PGx-guided dosing (that allowed for higher dosing), carriers of the *1B allele (homozygous or heterozygous) had significantly less withdrawal symptoms compared to patients on SOC dosing (*P* = 0.0294). No significant differences were observed with *CYP3A5* genetic variants or the use of unauthorized substances. The researchers concluded that *CYP3A4* genetic variants can impact clinical outcomes, and therefore, PGx-guided dosing should be implemented in buprenorphine therapeutics, especially for African American patients.

Using a sample from the previously mentioned START trial (*n* = 764/1,267) composed of European Americans (*n* = 599), African Americans (*n* = 79), and other ethnicities (*n* = 96), a different study evaluated variants in six pharmacokinetic genes (i.e., *CYP1A2*, *CYP2B6*, *CYP2C19*, *CYP2C9*, *CYP2D6* and *CYP3A4*) on dropout rate and mean dose^[29]^. No significant associations were identified for any variants of these genes in either the buprenorphine or methadone treatment arm.

Blanco *et al*.^[45]^ examined the effects of both the *UGT2B7* 802C>T (rs7439366) polymorphism and the *CYP3A4* 290A>G (rs2740574) polymorphism on analgesic response to buprenorphine. As previously mentioned, the study included 93 patients with critical limb ischemia who were treated with transdermal buprenorphine for pain. In this study, patients who were AA homozygotes for the *CYP3A4* gene showed the best response to analgesic treatment (*P* = 0.003), but no association was identified between the *UGT2B7* polymorphism and analgesic response to buprenorphine.

### Discussion

Buprenorphine dose adjustments based on *CYP3A4* functional status are the only PGx-guided interventions that have been implemented and evaluated to date. Findings from Ettienne *et al*.^[65,67]^ suggest that dosing patients by *CYP3A4* phenotype can improve treatment outcomes. Patients with an increased metabolic rate (i.e., EM and UM) may need higher doses to prevent withdrawal symptoms and maintain opioid abstinence. However, to date, these improvements have only been observed in African Americans. The sample from the START trial was mostly composed of European Americans (*n* = 599/764) and did not show significant associations between any metabolic gene variant and mean dose or dropout rate. Of note, results did trend towards a potential association between *CYP3A4* status and dropout rate, when the buprenorphine and methadone groups were combined. The authors hypothesized that a possible reason for the lack of significance was the low frequency of patients with PM or IM phenotypes in their study population with Western European ancestry. Again, further research with larger and more diverse patient populations is needed to assess the clinical benefits of *CYP3A4-*guided dosing (or other metabolic genes) for buprenorphine OUD treatment.

## PHARMACOGENES: ADVERSE EFFECTS

Genetic variations can not only influence treatment efficacy, dosing requirements and withdrawal incidence but adverse effects as well. Multiple studies involving methadone-treated patients have found associations between the rate and intensity of adverse effects with several pharmacogenes^[38]^. However, only one study has included buprenorphine in their evaluation of genetic variants and adverse effects. A prospective study analyzed variants in *OPRM1*, *OPRD1*, *COMT*, *ARRB2* and *ABCB1* on patients participating in an opioid deprescription program with buprenorphine patches^[68]^. The patient population was composed of Europeans with non-cancer chronic pain (*n* = 88, 64% females), who had been taking prescription opioids for more than 6 months, and were diagnosed with OUD at the start of the study. AA genotype at the *OPRM1* variant s1799971 was associated with a higher incidence of nausea (*P* = 0.034) and gastrointestinal adverse events (*P* = 0.031) when compared with AG/GG genotypes combined. Patients with the CT genotype at the *OPRD1* variant rs2234918 experienced less sexual dysfunction than TT/CC genotypes combined (*P* = 0.001). At the *COMT* variant rs4680, the AG genotype was associated with a lower incidence of loss of libido (*P* = 0.003) and skin redness (*P* = 0.003), while the AA genotype was associated with a higher incidence of vomiting (*P* = 0.003). Patients with the TT genotype at the *ARRB2* variant rs1045280 were less likely to experience loss of libido (*P* = 0.021) and dry skin (*P* = 0.024) than CC/CT genotypes. No significant differences were found with *ABCB1* variants.

### Discussion

To date, the study by Muriel *et al*.^[68]^ provides the only evidence on pharmacogenomic determinants of adverse events for patients with OUD treated with buprenorphine. Several factors, however, make interpretation of the study difficult. One key limitation in understanding the specific relation to buprenorphine is the inclusion of other opioids. The deprescription process included opioid tapering with the addition of tramadol, plus some patients were transitioned to fentanyl patches instead of buprenorphine (the authors do not report what percentage of patients were treated with buprenorphine patches compared to fentanyl patches). In addition, there were conflicting results between genotypes and the frequency of adverse events, when comparing baseline and final visits. For example, the AG genotype at *COMT* variant rs4680 was associated with less skin redness at baseline, but significantly more (than AA/GG genotypes) at the end of the program (*P* = 0.007). In summary, individual genotypes have the potential to contribute toward the rate and intensity of adverse events in OUD patients treated with buprenorphine. However, more research is needed to better understand the influence of genetic variants on adverse effects.

## CONCLUSION

The prevalence of OUD has reached unparalleled numbers in the last decades, increasing the rates of overdose fatalities and socioeconomic burden. Most of these patients do not receive proper treatment^[4]^, and furthermore, the lack of guidelines for individualized pharmacotherapy may limit the therapeutic benefits of medication-assisted treatment. Patient-specific factors can influence treatment response, but to date, clinicians are not equipped to use this data for therapy optimization. Understanding the pharmacogenomic variables that predict reductions in illicit opioid use, treatment adherence, and incidence of withdrawal symptoms would enhance clinical decisions.

For many years, methadone has been the primary option for medication-assisted treatment for OUD. However, buprenorphine may present a safer and more effective alternative for some patients based on comorbidities (e.g., mental health disorders) and genetic profile. Even though pharmacogenomic studies have accelerated in recent years, research involving PGx-guided strategies for OUD treatment has been limited, especially with buprenorphine. Nonetheless, the available data suggests significant relationships between some pharmacogenes and buprenorphine response [Table 1]^[69]^. Evidence is strongest for variants in opioid receptor pharmacogenes (*OPRD1*) and metabolic pharmacogenes (*CYP3A4*), but still limited with regards to dopaminergic pharmacogenes. Of particular clinical value might be genetic markers with ability to potentially direct treatment by identifying patients who may respond favorably to buprenorphine, but poorly to methadone and vice versa.

In conclusion, further research is needed to better understand how pharmacogenomic factors may be proactively used to improve treatment outcomes. As the vast majority of data that currently exists regarding pharmacogenomic determinants of buprenorphine response has been observational, the benefits of incorporating these factors into clinical treatment decisions remain theoretical. Thus, to date, there are no FDA-approved or Clinical Pharmacogenetics Implementation Consortium (CPIC®) recommendations for directing OUD therapy on the basis of pharmacogenomic testing. Hopefully, prospective clinical trials will better elucidate the role of PGx-guided treatment strategies. Additional research opportunities also exist in larger genome-wide association studies to identify novel genetic variants, investigating other potentially relevant candidate pharmacogenes (e.g., perhaps those affecting the ORL-1/NOP receptors), and research to better understand how other clinical factors such as treatment adherence interact with and mediate genetic factors. Lastly, as has been the case with most genetic research, greater diversity is needed in this field to ensure that the clinical gains from this research are broadly beneficial to all patients. Nevertheless, the development of PGx-guided strategies has the potential to reduce opioid misuse, improve clinical outcomes, and save healthcare resources.

## Figures and Tables

**Table 1.** Buprenorphine pharmacogenomic studies and findings

Ethnicity & Sex	Outcomes	Genes	Variant/allele	Findings	Ref.	Level of evidence^1^
Mixed, American (*n* = 410, 71% males) (74% European American) (Subset of START trial)	Mean dose, dropout rate	*OPRM1* *SLC6A4* *HTR2A* *ADRA2A* *COMT* *CYP1A2 CYP2B6 CYP2C19 CYP2C9 CYP2D6 CYP3A4*	rs1799971 5-HTTLPR rs6311 rs1800544 rs4680 Many^2^	No significant association found	[29]	Not available
European American (*n* = 299, 73% males) (subset of START trial)	Urine drug screens for opioids	*OPRM1*	Haplotype: rs671531 rs558948 rs645027 rs10485058	No significant association found	[30]	Not available
European American (*n* = 291, 73% males) *vs.* African American (*n* = 41, 66% males) (Subset of START trial)	Urine drug screens for opioids	*OPRD1*	rs678849 rs529520 rs581111 rs1042114 rs10753331 rs2234918	No significant associations observed amongst European Americans In the African American subset, a significant association was observed for rs678849, where carriers of the T-allele (CT/TT) were less likely to have opioid-positive drug screens, compared to the CC genotype. No significant associations were found with other *OPRD1* variants	[31]	*OPRD1* rs678849: Level 2B rs529520: Level 3 rs581111: Level 3 Other variants: Not available
Spanish (*n* = 93, 77% males)	Analgesic response to transdermal buprenorphine	*OPRM1* *UGT2B7* *CYP3A4*	rs1799971 rs7439366 rs2740574	No significant association was found for *OPRM1* or *UGT2B7* variants For CYP3A4 rs2740574, a significant association was found, where carriers of the AA genotype achieved increased pain relief	[45]	*CYP3A4* rs2740574: Level 3 Other gene-variants: Not available
European ancestry (*n* = 88, 64% females)	Response to opioid deprescription program^3^, incidence of adverse effects	*OPRM1* *OPRD1* *COMT* *ARRB2* *ABCB1*	rs1799971 rs2234918 rs4680 rs1045280 rs1045642	No associations were found between any variant and program response For *OPRM1* rs1799971, a significant association was found, where carriers of the G-allele (AG/GG) required higher MEDD for analgesic control and prevention of withdrawal symptoms For *OPRM1* rs1799971, AA genotype was associated with a higher incidence of nausea and gastrointestinal adverse events when compared with AG/GG genotypes For *OPRD1* rs2234918, CT genotype was associated with a lower incidence of sexual dysfunction than TT/CC genotypes For *COMT* rs4680, AG genotype was associated with a lower incidence of loss of libido and skin redness. AA genotype was associated with a higher incidence of vomiting *Vot ARRB2* rs1045280, TT genotype was associated with a lower incidence of dry skin and loss of libido when compared with CC/CT genotypes No significant differences were found with ABCB1 variants	[46,68]	*OPRM1* rs1799971: Level 3 *OPRD1* rs2234918: Level 3 *COMT* rs4680: Level 3 *ARRB2* rs1045280: Level 3 ABCB1 rs1045642: Not available
African American (*n* = 55, 84% males)	Urine drug screens for opioids	*OPRD1*	rs678849	A significant association was found, where carriers of the T-allele (CT/TT) were less likely to have opioid-positive drug screens, compared to the CC genotype	[48]	*OPRD1* rs678849: Level 2B
European American Males (*n* = 218) *vs.* Females (*n* = 81) (Subset of START trial)	Urine drug screens for opioids	*OPRD1*	rs581111 rs529520 rs1042114 rs678849 rs10753331 rs2234918	No significant associations observed amongst males. In the female subset, a significant association was observed for rs581111 and rs529520 For rs581111, female carriers of the A-allele (AG/AA) were more likely to have opioid-positive drug screens, compared to the GG genotype For rs529520, female carriers of the AA genotype were more likely to have opioid-positive drug screens, compared to the CC genotype. (AC genotype not significantly different than AA genotype)	[49]	*OPRD1* rs581111: Level 3 *OPRD1* rs529520: Level 3 Other variants: not available
Western European (*n* = 107, 81% males)	Responders *vs.* non-responders^4^	*OPRK1* *SLC6A3/DAT1*	rs1051660 3’UTRVNTR^5^	No significant association was found for the *OPRK1* variant For*SLC6A3/DAT1,* presence of the 11-repeat allele was significantly associated with the responder status. Presence of the 10-repeat allele was higher in non-responders (64.9%), but no significant association was observed	[55]	Not available
Australian (*n* = 25, 68% females) (88% European ancestry)	Treatment outcomes^6^, withdrawal incidence^7^, direct opioid effects^8^	*DRD2*	Taql A polymorphism (A1)	No significant association found	[60]	Not available
African American (*n* = 1 male)	Urine drug screens for unauthorized substances	CYP3A4	*1/*1B	As a CYP3A4*1B carrier, the patient was classified as an ultrarapid metabolizer (UM). Patient needed a dose of 32 mg to maintain abstinence from other unauthorized substances	[65]	CYP3A4*1B: Level 3
African American (*n* = 111, 76% males)	Dosing (SOC *vs.* PGx)^9^, withdrawal incidence, urine drug screens	CYP3A4 CYP3A5	N/A (phenotype)	No significant association found between *CYP3A5* and any of the measurements A significant association was found, where carriers of at least one copy of CYP3A4*’IB (UM), were less likely to experience withdrawal symptoms with PGx dosing PGx dosing was significantly associated with lower incidence of withdrawals, compared to SOC dosing	[67]	CYP3A4*1B: Level 3 CYP3A5: not available

1 Level of evidence based on Pharmacogenomics Knowledgebase (PharmGKB) criteria^69^ and current as of May 31, 2020. Available at https://www.pharmgkb.org/. 1A: Annotation for a variant-drug combination in a CPIC or medical society-endorsed PGx guideline, or implemented at a PGRN site or in another major health system. 1B: Annotation for a variant-drug combination where the preponderance of evidence shows an association. The association must be replicated in more than one cohort with significant P-values, and preferably will have a strong effect size. 2A: Annotation for a variant-drug combination that qualifies for level 2B where the variant is within a VIP (Very Important Pharmacogene) as defined by PharmGKB. The variants in level 2A are in known pharmacogenes, so functional significance is more likely. 2B: Annotation for a variant-drug combination with moderate evidence of an association. The association must be replicated but there may be some studies that do not show statistical significance, and/or the effect size may be small. 3: Annotation for a variant-drug combination based on a single significant (not yet replicated) study or annotation for a variant-drug combination evaluated in multiple studies but lacking clear evidence of an association. 4: Annotation based on a case report, non-significant study or in vitro, molecular or functional assay evidence only;

2 CYP variants included in Crist et al.^[29]^ 2018: CYP1A2: −3860G>A, −2467T>delT, −739T>G, −729C>T, −163C>A, 125C>G, 558C>A, 2116G>A, 2473G>A, 2499A>T, 3497G>A, 3533G>A, 5090C>T, 5166G>A, 5347C>T; CYP2B6: *1, *4, *6, *9; CYP2C19: *1, *2, *3, *4, *5, *6, *7, *8, *17; CYP2C9: *1, *2, *3, *4, *5, *6; CYP2D6: *1, *2, *2A, *3, *4, *5, *6, *7, *8, *9, *10, *11, *12, *14, *15, *17, *41; CYP3A4: *1, *13, *15A, *22;

3 non-responder = any of the following: (1) the patient dropped out of the individualized treatment program; (2) the diagnosis of prescription opioid dependence persisted according to DSM-5 criteria; (3) aberrant opioid use behavior persisted; or (4) the patient did not achieve at least a 30% reduction of the morphine equivalent daily dose (MEDD). Participants who did not meet these criteria were classified as responders. Within the responder group, a high responder subgroup was defined as patients who achieved a reduction of more than 50% of baseline MEDD;

4 non-responder = Any of the following: (1) early dropout from buprenorphine treatment and relapse to heroin (within the first 12 weeks); (2) continuous use of heroin during the treatment period (33% or more of urinalyses positive for morphine or cocaine metabolites); (3) severe behavioral or psychiatric problems in coincidence with buprenorphine treatment (aggressiveness episodes, severe mood problems, depression, delusions) with consequent switch to methadone or drug-free treatment; and (4) misbehavior concerning buprenorphine assumption (simulation of the assumption, diversion) and program discontinuation. Participants who did not meet these criteria were classified as responders;

5 variable number of tandem repeats (VNTR) in the 3′ untranslated region of exon 15;

6 Treatment outcomes = Self-reported illicit opioid use for last month, plasma morphine concentrations, and urine drug screen results;

7 Withdrawal = insomnia, muscle/bone/joint pains, nausea, craving, and reports of (MAT dose) “not holding”;

8 direct opioid effects = constipation, dry mouth, and itchy skin/nose;

9 dosing: standard of care (SOC) vs. pharmacogenomic-guided (PGx)
